# Association between engagement in-care and mortality in HIV-positive persons

**DOI:** 10.1097/QAD.0000000000001373

**Published:** 2017-03-01

**Authors:** Caroline A. Sabin, Alison Howarth, Sophie Jose, Teresa Hill, Vanessa Apea, Steve Morris, Fiona Burns

**Affiliations:** a Research Department of Infection and Population Health, University College London; b National Institute for Health Research Health Protection Research Unit (NIHR HPRU) in Blood Borne and Sexually Transmitted Infections at University College London; c Barts Health NHS Trust; d Department of Applied Health Research, University College London; e Royal Free London NHS Foundation Trust, London, UK.

**Keywords:** cohort studies, engagement, HIV, mortality, performance measures, retention

## Abstract

**Objective:**

To assess associations between engagement in-care and future mortality.

**Design:**

UK-based observational cohort study.

**Methods:**

HIV-positive participants with more than one visit after 1 January 2000 were identified. Each person-month was classified as being in or out-of-care based on the dates of the expected and observed next care visits. Cox models investigated associations between mortality and the cumulative proportion of months spent in-care (% IC, lagged by 1 year), and cumulative %IC prior to antiretroviral therapy (ART) in those attending clinic for more than 1 year, with adjustment for age, CD4^+^/viral load, year, sex, infection mode, ethnicity, and receipt/type of ART.

**Results:**

The 44 432 individuals (27.8% women; 50.5% homosexual, 28.9% black African; median age 36 years) were followed for a median of 5.5 years, over which time 2279 (5.1%) people died. Higher %IC was associated with lower mortality both before [relative hazard 0.91 (95% confidence interval 0.88–0.95)/10% higher, *P* = 0.0001] and after [0.90 (0.87–0.93), *P* = 0.0001] adjustment. Adjustment for future CD4^+^ changes revealed that the association was explained by poorer CD4^+^ cell counts in those with lower %IC. In total 8730 participants under follow-up for more than 1 year initiated ART of whom 237 (2.7%) died. Higher values of %IC prior to ART initiation were associated with a reduced risk of mortality before [0.29 (0.17–0.47)/10%, *P* = 0.0001] and after [0.36 (0.21–0.61)/10%, *P* = 0.0002] adjustment; the association was again explained by poorer post-ART CD4^+^/ viral load in those with lower pre-ART %IC.

**Conclusions:**

Higher levels of engagement in-care are associated with reduced mortality at all stages of infection, including in those who initiate ART.

## Background

The widespread use of effective antiretroviral therapy (ART) has led to a dramatic reduction in morbidity and mortality among people living with HIV [1]. ART is also recognized as an effective means of reducing HIV transmission [2]. However, the individual and public health benefits of ART can only be achieved if people living with HIV are aware that they are HIV positive, have linked into care, and have sustained engagement with care thereafter.

Unpublished data from London HIV clinics indicate that outpatient nonattendance rates can be as high as 25% (Iain Reeves, personal communication). Poorer health outcomes, including failure to suppress HIV viraemia, increased drug resistance and suboptimal CD4^+^ cell count responses, are reported among people living with HIV who engage poorly with care [3–8]. In the United Kingdom, missed outpatient appointments have significant resource implications [9], and the importance of continuity of care for reducing costs has been recognized in several disease areas [10–12]. In the HIV setting, retaining HIV-positive persons in care may also reduce new infections, treatment costs, and deaths [13,14].

To explore patterns of engagement in HIV care, it is essential to adopt a valid and reliable measure, and yet there is no gold standard measure of engagement in HIV outpatient care. Researchers have assessed retention in care in several ways, with definitions commonly based on the quality indicators proposed by the US Health Resources and Services Administration HIV/AIDS Bureau (HRSA HAB), the US Institute of Medicine (IOM), or the US Department of Health and Human Services (DHHS) [6,15,16]. These measures have their own strengths and weaknesses [17], but none of them take into account the fact that frequency of attendance is related to changes in ART and health status and may also be affected by external forces or changes in clinic policy. In the United Kingdom, for example, British HIV Association (BHIVA) guidelines from 2011 indicated that HIV-positive persons should be seen within 2–4 weeks of starting ART and every 3–6 months thereafter for routine monitoring if they were considered ‘stable’ [18]; recently updated guidelines suggest that monitoring frequency can now be reduced to 6 monthly for those on ART with stable viral suppression [19].

The REACH study (Exploring patterns of Retention and Engagement Across specialised Care services for HIV patients in the United Kingdom) set out to develop a dynamic measure of engagement in-care that would be sensitive to changes in an individual's status over time [20]. Using this measure, we demonstrated that individuals were deemed to be in-care for 83.9% of the total follow-up time. To date, however, we have not investigated whether this measure is able to identify a group of individuals at high risk of clinical progression. One of the main limitations of analyses that investigate associations between engagement in-care and clinical outcomes is the potential for reverse causality whereby those who are sickest may attend for care more frequently as their health deteriorates, thus creating an artificial association between higher levels of engagement and an increased risk of clinical progression. Thus, the objective of the present analysis was to use data from the UK Collaborative HIV Cohort (UK CHIC) study to investigate associations between our measure of engagement in-care and mortality, while reducing any potential impact of reverse causality.

## Methods

The UK CHIC Study was initiated in 2001 and collates routine data on HIV-positive persons attending many of the UK's largest HIV clinical centres. The project was approved by a Multicentre Research Ethics Committee and local ethics committees. HIV-positive individuals are included in the study provided they have attended one of the collaborating centres at any time since 1 January 1996 and are aged at least 16 years [21]. The data set used for the present study is based on data submitted from 19 clinics with data collection up to 31 December 2012. The work was conducted as part of the National Institute for Health Research Health Protection Research Unit (NIHR HPRU) in Blood-Borne and Sexually Transmitted Infections (UCL in collaboration with London School of Hygiene and Tropical Medicine), a partnership with Public Health England. The UK CHIC records are linked anonymously to mortality data collected as part of national surveillance programmes conducted by Public Health England.

Our measure of engagement in-care has been described [20]. In brief, in the absence of complete data on clinic attendances, we used CD4^+^ cell counts, viral loads, haemoglobin measures and ART start/switch dates as markers of clinic attendance. As people often return for repeat laboratory tests over a short period of time to confirm unexpected findings, resulting in clusters of attendances around a single ‘index’ date, we grouped attendances into ‘care episodes’, defined as months where at least one visit occurred. For each care episode, we established the lowest CD4^+^ cell count measured in that month (and the change from the previous value), the highest HIV viral load, the person's ART status and identified whether the person had received a recent HIV diagnosis, and used this information to establish the likely month of the next scheduled visit using our algorithm. The date of the next observed care visit determined whether the person had attended at any time in the period leading up to the expected date. If so, the person-months up to the observed visit were all classified as being in-care; if not, the person-months up to the date of the expected visit were classified as in-care (as the person's attendance had not, at that point, deviated from what was expected) whereas the subsequent person-months until the observed visit were classified as out-of-care. Each person-month between consecutive visits was thus classified as being in or out-of-care.

We included all people who attended a participating UK CHIC clinic on at least one occasion between 1 January 2000 and 31 December 2012. We used Cox models to assess the association between mortality and the cumulative proportion of months a person had been in-care (%IC) and the cumulative proportion of months a person was in-care prior to ART.

For analyses of the association within the full cohort, person follow-up started at study entry and ended at the earliest of death or six months after the person's last clinic attendance. Each person's total follow-up period was split into consecutive monthly intervals and the cumulative proportion of previous months she/he had been in-care at the start of each month was calculated (%IC). In this way, we were able to include %IC as a continuous time-updated covariate in regression models. To reduce the potential for our analyses to be affected by reverse causality, all measures of %IC were lagged by 12 months to separate the assessment of engagement and the outcome by a period of 1 year. Thus, our estimated relative hazard for %IC will describe the relationship between %IC and clinical events that occur at least 1 year into the future. Note that this approach will necessarily restrict analyses to those who had attended clinic for more than 1 year. In our primary analyses we adjusted for the demographic factors: age, year, sex, mode of HIV acquisition and ethnic group (fixed covariates). This was followed by additional adjustment for receipt of ART as a binary time-updated covariate. We next adjusted for the latest CD4^+^ cell count, as a continuous time-updated covariate and lagged by 12 months, to investigate whether any association seen with %IC was explained by the fact that those with the lowest %IC values already had poorer CD4^+^ cell counts at the time of assessment of engagement. Finally, we adjusted for the unlagged values of CD4^+^ to explore whether any residual association between %IC and clinical outcomes was mediated by lower CD4^+^ cell counts over the following 12 months. We did not adjust for the latest viral load given the expected collinearity between this and our ART variable.

For analyses of the subgroup of patients who started ART, we calculated the %IC prior to ART in the subset of people who initiated ART and who had been under follow-up at the clinic for at least a year prior to ART start. Pre-ART %IC was stratified into six groups (<50%, 50–69.9%, 70–79.9%, 80–89.9%, 90–99.9%, and 100%) which were chosen for ease of clinical interpretation and to ensure that each group was of sufficient size to permit robust analyses. Cox models then considered the association between pre-ART %IC (as a fixed baseline covariate) and mortality after ART initiation; follow-up started at ART initiation and ended at the earliest of 6 months after the person's last visit or death. We first adjusted for age, year, sex, mode of HIV acquisition and ethnic group, then type of ART received [protease inhibitor based, NNRTI (nonnucleoside reverse transcriptase inhibitor) based, other regimen (including those on both a protease inhibitor and NNRTI)], then the CD4^+^ cell count and viral load at ART start (fixed covariates) and then finally the latest CD4^+^ cell count and viral load (as time-updated covariates) measured after ART start. As before, these latter analyses investigate whether any associations between pre-ART %IC and post-ART mortality can be explained by poorer CD4^+^/viral load responses on ART. Note that as we considered pre-ART %IC and outcome post-ART, then reverse causality is unlikely to be of major concern in this analysis.

## Results

A total of 44 432 UK CHIC participants were included in the initial analysis (Table 1). Women represented 27.8% of the sample. Half were white (53.3%), one third were black African (28.9%), 8.7% were of other ethnicity, and 9.2% had unknown ethnicity. Around half had acquired HIV through sex between men (50.5%), with 39.1% acquiring HIV through sex between men and women, 3.0% through injection drug use and the remaining 7.4% through other or unknown routes. The median age at entry into the study was 36 years (interquartile range (IQR) 30–42] and the median date of follow-up start was December 2004 [range January 2000–October 2012). The median CD4^+^ cell count at start of follow-up was 355 (IQR 214–520) cells/μl.

**Table 1:** Characteristics of patients included in the two sets of analyses.

		All study participants at baseline	Study participants at ART^1^ start
N		44 432	8730
Sex (%)	Men	72.2	78.2
	Women	27.8	21.8
Age (years)	Median (IQR)	36 (30, 42)	37 (32, 43)
Exposure (%)	MSM	50.5	62.3
	Heterosexual	39.1	31.1
	IDU	3.0	2.9
	Other/unknown	7.4	3.7
Ethnic group (%)	White	53.3	63.4
	Black African	28.9	20.9
	Other	8.7	8.9
	Unknown	9.2	6.8
CD4^+^ cell count (cells/μl)	Median (IQR)	355 (214, 520)	280 (202, 368)

ART, antiretroviral therapy; IQR, interquartile range.

Over a median follow-up of 5.5 (IQR 2.0, 10.0) years, 6685 (15.1%) people developed a new AIDS event and 2279 (5.1%) died. Table 2 shows the association between engagement in-care and mortality, first without adjustment, then after performing an adjusted analysis. The estimate of the relative hazard after adjustment for fixed covariates and ART status demonstrates that higher engagement in-care is associated with less rapid progression to death when this is considered at least 1 year into the future. Adjustment for the lagged CD4^+^ cell count resulted in an attenuation of the association between %IC and mortality, demonstrating that a proportion of the association seen can be explained by the fact that those with lower %IC values also have lower CD4^+^ cell counts. Additional adjustment for the unlagged CD4^+^ cell counts led to further attenuation of the estimate towards 1, suggesting that in addition to poorer CD4^+^ cell counts at the time of %IC assessment, those with lower %IC values also maintained lower CD4^+^ cell counts over the subsequent 12-month period.

**Table 2:** Results from unadjusted and adjusted Cox regression analyses of associations between %IC and mortality: all patients, and patients starting antiretroviral therapy.

	Mortality
i) All patients	RH (95% CI)/10% higher %IC
Total number (%) deaths	2279 (5.1)
Adjustment for:	
None	0.91 (0.88, 0.95)
Fixed covariates	0.91 (0.88, 0.95)
+Receipt of ART (yes/no)	0.90 (0.87, 0.93)
+Latest CD4^+^ cell count (lagged)	0.96 (0.92, 1.00)
+Latest CD4^+^ cell count (unlagged)	1.00 (0.96, 1.04)
ii) Patients starting ART	RH (95% CI)/10% higher %IC
Total number (%) deaths	237 (2.7%)
Adjustment for:	
None	0.29 (0.18, 0.47)
Fixed covariates	0.31 (0.18, 0.51)
+Baseline ART regimen^a^	0.32 (0.19, 0.53)
+Baseline CD4^+^ cell count and viral load	0.36 (0.21, 0.61)
+Latest CD4^+^ cell count and viral load	0.74 (0.42, 1.30)

ART, antiretroviral therapy; CI, confidence interval; RH, relative hazard.

^a^Protease inhibitor based, nonnucleoside reverse transcriptase inhibitor based or other.

A total of 8730 individuals had been under follow-up at a participating clinic before starting ART (Table 1). Compared with the wider population group, those starting ART were more likely to be men, were slightly older, more likely to have acquired HIV through sex between men, of white ethnicity and had a lower CD4^+^ cell count.

At the time of starting ART, the median %IC was 85.7% (range 3.3–100.0%, IQR 65.5–95.7%); %IC was <50%, 50–70%, 70–80%, 80–90%, 90–99%, and 100% in 1282 (14.7%), 1239 (14.2%), 1014 (11.6%), 1591 (18.2%), 2092 (24.0%), and 1512 (17.3%), respectively. Table 3 shows associations between the pre-ART %IC and several selected demographic and clinical characteristics. Men were more likely than women to have spent a greater proportion of time in-care before starting ART. Other factors associated with a higher proportion of time spent in-care were HIV acquisition through sex between men, white ethnicity, a higher CD4^+^ cell count at start of ART, and initiation of ART with an NNRTI-based regimen.

**Table 3:** Associations between preantiretroviral therapy engagement in-care and selected demographic/clinical factors at start of antiretroviral therapy.

	*n* (%) of group	Men	MSM	White		Regimen	
%months IC prior to ART					CD4^+^ (cells/μl) Median	PI	NNRTI
	%	%	%	%		%	%
<50%	1282 (14.7)	73.1	46.2	53.5	250	32.1	60.8
50–70%	1239 (14.2)	76.0	59.5	60.9	259	25.3	66.4
70–80%	1014 (11.6)	77.7	62.8	62.1	280	25.5	67.5
80–90%	1591 (18.2)	80.1	65.6	64.9	283	26.2	67.1
90–99%	2092 (24.0)	79.3	66.4	65.6	290	23.0	68.6
100%	1512 (17.3)	81.0	68.6	70.3	299	21.4	70.0

ART, antiretroviral therapy; IC, in-care; MSM, men who have sex with men; NNRTI, nonnucleoside reverse transcriptase inhibitor; PI, protease inhibitor.

Over a total post-ART follow-up period of 4.3 (IQR 2.1, 6.8) years, 237 (2.7%) people who started ART died. Table 2 shows the association between pre-ART %IC and post-ART mortality, after progressively adjusting for covariates as before. As in the previous analyses, the strong association between pre-ART %IC and post-ART mortality that was apparent in unadjusted analyses and analyses that control for the baseline covariates was substantially attenuated towards 1 after further adjustment for post-ART CD4^+^ cell count and viral load profiles.

## Discussion

Using a flexible approach to measuring engagement in-care that can be adapted to the changing health status of the individual and to local clinic policies, we have demonstrated that higher levels of engagement in HIV care are associated with reduced mortality at all stages of infection, both in individuals who are receiving ART and those who are not. Our findings suggest that this association is largely explained by poorer CD4^+^ cell count profiles in those with suboptimal engagement in-care.

### Definitions of engagement in-care

Although the importance of a high rate of engagement is well recognised, definitions vary widely between studies [22]. Within the largely North American literature on the topic, studies have predominantly used one of the measures utilized by the US DHHS/HRSA HAB, the IOM or some modification, all of which assess ‘visit constancy’ over a period of time. For example, the DHHS/HRSA HAB indicator defines individuals as being engaged in-care if they have at least one visit in each of four consecutive 6-month periods, with visits separated by more than 60 days [5,17,23–25]. The IOM core indicator classifies individuals as engaged in-care if they have at least two visits in a 12-month period, where each visit is separated by more than 90 days [7,16,17,23,25–28], although adaptations that require only 60 days of separation are also used [29,30]. Other measures of visit constancy consider the proportion of a set number of consecutive time intervals in which patients attend at least one scheduled appointment [17,26,27,31] or consider a patient to be engaged in-care if there is no gap in care more than 6 months [17,26,27,32].

Although measures of visit constancy provide information on visits that are kept, they provide little information on missed visits. Measures of ‘visit adherence’ have therefore also been proposed that classify individuals according to the number or proportion of scheduled visits that have been missed over a period [26,31]. Collection of data on unscheduled missed visits may, however, be challenging for many clinics, particularly if visits are not recorded as ‘missed’ if they are rescheduled in advance. Such measures may also be difficult to implement at a region-wide level or in large collaborative studies where computer systems may vary. For pragmatic reasons, therefore, simpler measures of engagement in-care are often preferred [33–38].

This variation between studies introduces complexity into any comparison of rates of engagement in different settings. Mugavero [26] demonstrated considerable variability between six measures of retention in terms of the proportion of patients who were considered as retained in-care. Although each measure was strongly correlated with viral load suppression, the correlation between the various measures of retention was as low as 0.16.

### Associations with clinical and virological outcomes

Several studies have described the association between engagement in-care and clinical and virological outcomes. In the South Carolina, enhanced HIV/AIDS Reporting System (eHARS) surveillance database [5], mean decrease in viral load and increase in CD4^+^ cell count were both greater in those with optimal retention in-care, and associations with poorer engagement in-care and mortality have been reported by several groups [5,6,39]. In a study from the Centers for AIDS Research (CEFAR) Network of Integrated Clinical Systems (CNICS) [23], failure to achieve the IOM and DHHS indicators was associated with increased mortality; among those classified as retained in-care by either indicator, however, having more than two missed visits was also associated with an additional increase in mortality risk. The authors noted that lifestyle or behavioural factors that may have increased the risk of a person missing a clinic appointment may also have been associated with an increased risk of mortality. Findings from the ALIVE study [32], in which a significant association between reporting no HIV care visit in the past 6 months and virological failure was lost after adjustment for factors that included predictors of lapses in care, would support the concern that reported associations between engagement in-care and virological suppression may not necessarily be causal. Findings of a stronger association between retention in-care and viral suppression among those with lower CD4^+^ cell counts and in younger adults [17,27], raise the interesting prospect that measures of engagement in-care should be interpreted in light of other individual-level characteristics.

### Limitations of current measures of engagement

Several limitations of the currently used measures of engagement in-care have been noted [25]. First, although most approaches generally consider engagement in a cross-sectional manner, engagement in-care is an evolving process, with individuals often moving in or out of care as their personal circumstances and health change. For example, Colasanti reported that while 12-month retention and virological suppression rates at the Infectious Disease Program of the Grady Health System in Atlanta were both high, only 49% of participants had maintained continued retention and only 39% continuous virological suppression for 36 months [15]. Furthermore, clinic monitoring policies are also changing, in response to improved outcomes on ART and increasing patient numbers. Our proposed approach to defining engagement in-care reflects such changes at both an individual and clinic level.

Second, associations between calendar year and engagement in-care are often subject to bias. It may be difficult to differentiate between permanent loss to follow-up and transient interruptions in-care among patients followed in more recent periods because of insufficient follow-up [40], and though it may be tempting to attribute improvements in engagement in-care rates to the increased use of interventions to reduce disengagement, apparent improvements may simply reflect the fact that those who are diagnosed and/or start ART in later follow-up periods may have had less opportunity to miss a clinic appointment.

Third, most measures of engagement in-care fail to take consideration of the fact that individuals may seek care from other providers. The implications of such individuals for the overall care cascade are unclear – in Southern Alberta, patients who left the HIV care program but who returned at a later date had deterioration in both CD4^+^ cell counts and new AIDS events compared with those who had never left the program, in whom CD4^+^ cell counts increased [41]. In contrast, transfers into the care centre from outside the area had greater engagement in-care than local patients [33]. At a population level, the choice of denominator can be crucial for estimates: engagement in-care from King County, Washington, ranged from 66–81% depending on the definition of the denominator population [42].

Finally, although engagement in-care would ideally be measured through face-to-face clinical encounters, many studies have used laboratory markers of disease progression (e.g. CD4^+^ cell counts and viral loads) as surrogates for clinic encounters [43–47]. Reflecting the fact that the UK CHIC study is a large study which uses routinely collected electronic data only, our proposed algorithm also uses a combination of laboratory and clinical data as a surrogate. The potential for this approach to underestimate engagement in-care has been assessed, with studies generally reporting that while laboratory markers are able to discriminate between persons retained and not retained in-care, absolute engagement rates are often lower when estimates are based on laboratory data [44–46]. In contrast, in a small study of 99 newly diagnosed patients in Louisiana, Halperin [28] reported that the use of laboratory markers as a proxy for clinical visits tended to overestimate currently accepted definitions of engagement in-care.

### Strengths and limitations of the present study

Our analyses benefit from a large, prospectively compiled, clinical data set with longitudinal data on CD4^+^ cell counts, viral loads, and ART use. By separating our assessment of engagement from clinical outcomes by 12 months, we were also able to reduce the impact of reverse causality, whereby individuals who are sicker may attend for care more frequently than those who are well (although we recognize that some residual reverse causality bias may remain). However, some limitations of our study should be noted. By taking this approach, we are unable to assess the impact of engagement in care on outcomes within the first 12 months of follow-up for an individual, and our analyses may not be generalizable to those under care for periods of less than 12 months. We attempted to investigate whether the association seen could be mediated through poorer CD4^+^ profiles both at the time of assessment of engagement in-care as well as over the subsequent period. However, these analyses will be incomplete if follow-up CD4^+^ cell counts and viral loads are more likely to be missing in those with poorer engagement in-care. Although our algorithm can be adapted to other settings (e.g. resource-limited settings where laboratory monitoring is less frequent), generalizability to those settings is uncertain. Finally, we recognize that our algorithm does not capture additional information that might modify a clinician's decision about the timing of the next visit (e.g. psychosocial factors).

### Summary

In summary, we have shown that, among patients attending for care for at least a year, a combination of routinely collected clinical and laboratory data is able to identify, through engagement patterns, individuals at increased risk of subsequent mortality both before and after starting ART.

## Acknowledgements

UK CHIC is funded by the UK Medical Research Council (Grant numbers G0000199, G0600337, G0900274 and M004236). The REACH study was supported by the National Institute for Health Research's HS&DR Programme (project number 11/2004/50). The views expressed in this manuscript are those of the researchers and not necessarily those of the Medical Research Council or HS&DR Programme.

C.A.S., F.B., V.A. and S.M. were responsible for designing, obtaining funding for and setting up the REACH study; along with AH, they all conducted analyses to develop the REACH algorithm that is used in the present analysis. C.A.S. was responsible for designing, obtaining funding for and setting up the UK CHIC study, undertook all analyses for the present manuscript and prepared drafts of the manuscript. S.J. and T.H. provided input to the generation of all analysis datasets. All study authors contributed to initial discussions around the application of the REACH algorithm to the UK CHIC dataset, around the interpretation of study findings, to the development of the manuscript and all have critically reviewed and approved the final version of the submitted manuscript.

UK CHIC Steering Committee: Jonathan Ainsworth, Sris Allan, Jane Anderson, Abdel Babiker, David Chadwick, Valerie Delpech, David Dunn, Martin Fisher, Brian Gazzard, Richard Gilson, Mark Gompels, Phillip Hay, T.H., Margaret Johnson, S.J., Stephen Kegg, Clifford Leen, Fabiola Martin, Mark Nelson, Chloe Orkin, Adrian Palfreeman, Andrew Phillips, Deenan Pillay, Frank Post, Jillian Pritchard, C.A.S., Memory Sachikonye, Achim Schwenk, Anjum Tariq, Roy Trevelion, John Walsh.

UK CHIC Central Co-ordination: University College London (T.H., S.J., Andrew Phillips, C.A.S.); Medical Research Council Clinical Trials Unit at UCL (MRC CTU at UCL), London (David Dunn, Adam Glabay).

UK CHIC Participating Centres: Brighton and Sussex University Hospitals NHS Trust (M. Fisher, N. Perry, S. Tilbury, E. Youssef, D. Churchill); Chelsea and Westminster Hospital NHS Foundation Trust, London (B. Gazzard, M. Nelson, R. Everett, D. Asboe, S. Mandalia); King's College Hospital NHS Foundation Trust, London (F. Post, H. Korat, C. Taylor, Z. Gleisner, F. Ibrahim, L. Campbell); Mortimer Market Centre, University College London (R. Gilson, N. Brima, I. Williams); Royal Free NHS Foundation Trust/University College London (M. Johnson, M. Youle, F. Lampe, C. Smith, R. Tsintas, C. Chaloner, S. Hutchinson, C. Sabin, A. Phillips, T. Hill, S. Jose, A. Thornton, S. Huntington); Imperial College Healthcare NHS Trust, London (J. Walsh, N. Mackie, A. Winston, J. Weber, F. Ramzan, M. Carder); Barts and The London NHS Trust, London (C. Orkin, J. Lynch, J. Hand, C. de Souza); Homerton University Hospital NHS Trust, London (J. Anderson, S. Munshi); North Middlesex University Hospital NHS Trust, London (J. Ainsworth, A. Schwenk, S. Miller, C. Wood); The Lothian University Hospitals NHS Trust, Edinburgh (C. Leen, A. Wilson, S. Morris); North Bristol NHS Trust (M. Gompels, S. Allan); Leicester, University Hospitals of Leicester NHS Trust (A. Palfreeman, K. Memon, A. Lewszuk); Middlesbrough, South Tees Hospitals NHS Foundation Trust (D. Chadwick, E. Cope, J. Gibson); Woolwich, Lewisham and Greenwich NHS Trust (S. Kegg, P. Main, Dr Mitchell, Dr Hunter), St George's Healthcare NHS Trust (P. Hay, M. Dhillon); York Teaching Hospital NHS Foundation Trust (F. Martin, S. Russell-Sharpe); Coventry, University Hospitals Coventry and Warwickshire NHS Trust (S. Allan, A. Harte, S. Clay); Wolverhampton, The Royal Wolverhampton Hospitals NHS Trust (A. Tariq, H. Spencer, R. Jones); Chertsey, Ashford and St Peter's Hospitals NHS Foundation Trust (J. Pritchard, S. Cumming, C. Atkinson); Public Health England, London (V. Delpech); UK Community Advisory Board (R. Trevelion)

HPRU: This report is independent research supported by the National Institute for Health Research. The research was funded by the National Institute for Health Research Health Protection Research Unit (NIHR HPRU) in Blood Borne and Sexually Transmitted Infections at UCL and in collaboration with the London School of Hygiene and Tropical Medicine in partnership with Public Health England. The views expressed in this publication are those of the author(s) and not necessarily those of the NHS, the National Institute for Health Research, the Department of Health or Public Health England.

### Conflicts of interest

C.A.S. has received funding for the membership of Data Safety and Monitoring Boards, Speaker Panels, Advisory Boards and for preparation of educational materials from Gilead Sciences, ViiV Healthcare and Janssen-Cilag; S.J. has received funding for the preparation of lectures from Gilead Sciences; F.B. has received funding for consultancy, grants and travel from Gilead sciences.
